# Protein biomarkers of external root resorption: A new protein extraction protocol. Are we going in the right direction?

**DOI:** 10.1590/2176-9451.19.6.062-069.oar

**Published:** 2014

**Authors:** Giovanni Modesto Vieira

**Affiliations:** 1 University of Brasília, PhD resident in Medical Sciences, University of Brasília (UnB)

**Keywords:** Root resorption, Molecular diagnosis technique, Gingival crevicular fluid, Electrophoresis, Isoelectric focusing

## Abstract

**OBJECTIVE:**

The aim of this study is to determine a protocol of gingival crevicular fluid protein extraction used for the first dimension of 2-DE gels. It also aims at conducting a review on the current candidates for protein markers of this pathology, all of which may be used to prevent the disease.

**METHODS:**

Gingival crevicular fluid was collected from two groups of 60 patients each, with and without external root resorption. Samples were extracted by means of various methods of protein extraction. SDS-PAGE gels were used to assess the quality of the method which was subsequently tested during isoelectric focusing of 2-DE gels taken from samples of patients with and without the disease.

**RESULTS:**

Milli-Q ultrapure ice cold water, without precipitation for gingival crevicular fluid protein extraction, proved the method with greatest sharpness to detect protein bands. Additionally, it allowed two-dimensional electrophoresis to be performed.

**CONCLUSION:**

The new protein extraction protocol does not interfere in isoeletric focusing of 2-DE gels. Furthermore, it provides the greatest sharpness in detecting protein bands of SDS-PAGE gels. This will allow mapping and searching of new external root resorption markers, particularly due to the difficulty in carrying out molecular tests with the current candidates for protein markers.

## Abstract (pt)

**OBJETIVO:**

o objetivo desse trabalho foi determinar o protocolo de extração proteica do fluido crevicular gengival, que pudesse ser utilizado para a realização da primeira dimensão dos géis 2-DE, bem como fazer uma revisão dos atuais candidatos a marcadores proteicos dessa patologia que podem ser utilizados na prevenção dessa doença.

**MÉTODOS:**

foi coletado o fluido crevicular gengival de dois grupos de 60 pacientes, com e sem a reabsorção radicular externa. As amostras foram extraídas por diversos métodos de extração proteica e utilizados géis SDS-PAGE para aferir a qualidade do método, que posteriormente foi testado durante a realização da focalização isoelétrica dos géis 2-DE, de amostras de pacientes com e sem a patologia.

**RESULTADOS:**

a utilização de água Milli-Q gelada ultrapura, sem nenhuma precipitação para a extração proteica do fluido crevicular gengival, foi o método com maior nitidez das bandas proteicas, além de permitir a realização da eletroforese bidimensional.

**CONCLUSÕES:**

o novo protocolo de extração proteica não interfere na focalização durante a realização dos géis 2-DE, além de maior nitidez na resolução das bandas proteicas dos géis SDS-PAGE. Isso permitirá o mapeamento e busca de novos marcadores da reabsorção radicular externa, tendo em vista a dificuldade de realização de testes moleculares com os atuais candidatos a marcadores proteicos.

## INTRODUCTION

The high prevalence of inflammatory external root resorption (IERR) associated with orthodontic treatment (95 to 100%)^1^ poses the need to integrate basic research, supported by scientific evidence, with daily clinical practice in order to minimize the biological costs (IERR) of orthodontic treatment itself.

The expression of gingival crevicular fluid markers (GCF) is mainly used in Dentistry to estimate the immune response of the host to periodontal disease.^2^ The potential use of GCF markers proves a non-invasive technique employed to clinically track the activity of osteoclasts, bone remodeling and external root resorption occurring during orthodontic treatment.^2^

Evans *et al* 3 demonstrated the presence of gingival crevicular fluid proteins, particularly the dentin matrix protein (DMP-1), in patients undergoing orthodontic treatment. Since then, the study of these proteins has significantly deepened, and research correlating their presence with root resorption has markedly increased.^3^

Because they are part of the dental tissue, more specifically the dentin, these proteins are not routinely released into periodontal ligament spaces, unless active external root resorption is present.^4^

The search for IERR markers was intensified by the discovery of dentin-specific proteins (dentin phosphoprotein-DPP and dentin sialoprotein-DSP) which appeared as by-products of root resorption in the gingival crevicular fluid. They were analyzed by enzyme-linked immunosorbent assays (ELISA) by James Mah^5^ (DPP), and subsequently confirmed (DPP and DSP) by Laura Balducci et al^4^ by means of one-dimensional electrophoresis (SDS-PAGE), Western blot and ELISA; and Shalene *et al* 6 (DSP) also by Western blot and ELISA.^4^^,^ 5 ^,^ 6

In all these researches, protein extraction was performed through sodium phosphate buffer, which hinders visualization of protein bands in SDS-PAGE gel and its later use in proteomic techniques of higher resolution (two-dimensional electrophoresis-2DE) to precisely identify the proteins contained in the samples.

The present article aims at reporting the use of a new method of GCF protein extraction, which provides better visualization of samples and does not interfere in the isoelectric focusing of 2DE gels, as in the classical extraction technique. This new protocol will enable accurate mapping of proteins related to IERR. The present study also discusses whether the current candidates for dentin protein markers reported in the literature can actually be used as molecular diagnostic kits for the prevention of IERR sequelae in patients undergoing orthodontic treatment.

## MATERIAL AND METHODS:

## Sampling

**Figure 1.**
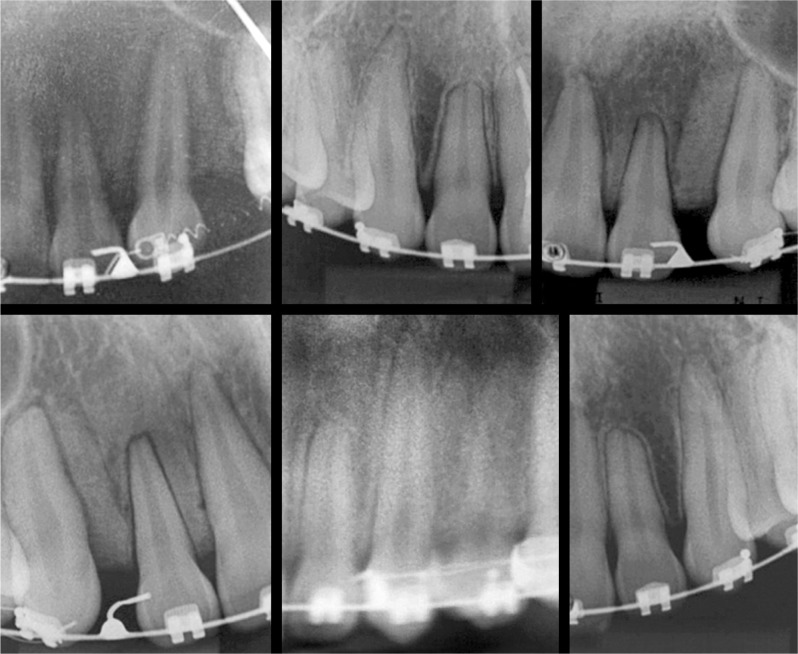
Periapical radiograph of patients comprising the mild to moderate IERR group.

The sample comprised 60 patients (22 men and 38 women) aged between 15 and 30 years old who did not have systemic disease, periodontal disease, gingivitis or tooth decay. In addition, they did not take any systemic medication. Patients were divided into two groups: Group 1 (control) comprising 30 patients who had been undergoing orthodontic treatment for at least six months without IERR being revealed by periapical radiographs; and Group 2 comprising 30 patients who had been undergoing orthodontic treatment for at least six months with mild to moderate IERR, according to the classification by Levander and Malmgren, as shown in radiographic examination^7^ (Fig 1).

## Gingival fluid collection

Sterile absorbent paper cones were used according to the method proposed by Burke et al^8^ and Bang et al.^9^

## Protein extraction

**Figure 2.**
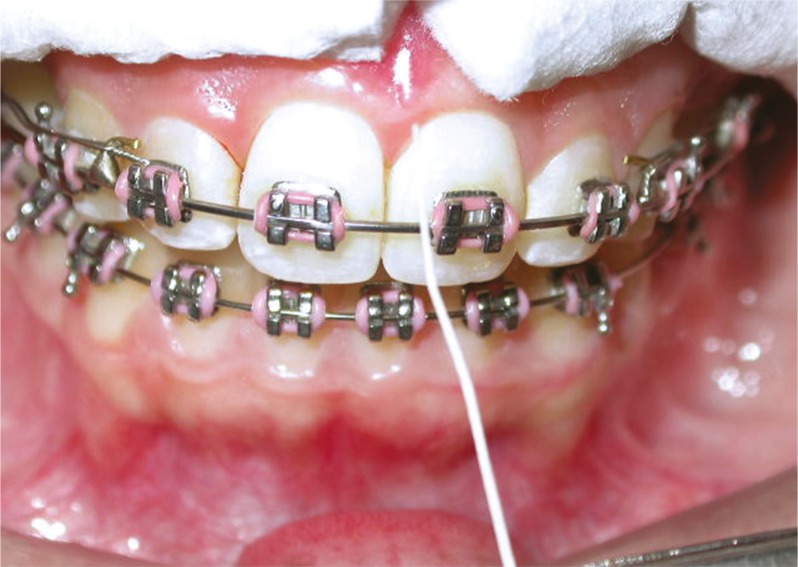
Patient subjected to prophylaxis followed by relative isolation with cotton rolls and the use of ejector. GCF sample collection with absorbent paper cones.

Protein extraction was performed without protein precipitation. The cones containing gingival fluid samples from both groups (Fig 2) were collected to form a pool of proteins from each group. A total of 100 µl of ultrapure ice cold water (Milli-Q RG, Millipore) and protease inhibitor (PMSF-phenyl methyl sulfonyl fluoride) were added to every pair of absorbent paper cones which were then centrifuged twice at 13.400 rpm for 5 minutes. The process was repeated and the supernatant with eluted proteins was lyophilized and stored for subsequent electrophoretic analysis.

**Figure 3.**
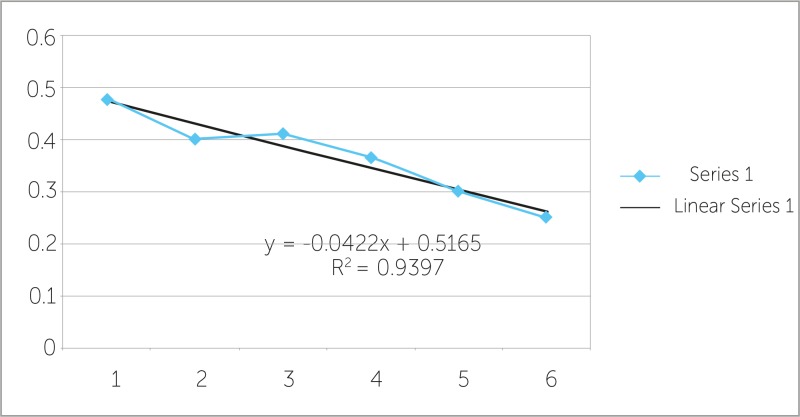
2-DE Quant kit curve depicting 12µg/µl and 8 µg/µl of proteins for samples with and without IERR

Protein quantification was carried out by the 2-DE Quant kit (Amersham biosciences-GE Healthcare), following the manufacturer's instructions (Fig 3). The major advantage of this technique lies in the application of copper ions which bind to the main protein chain. It differs from conventional protein quantification techniques that do not use this ion and bind to arginine and hydrophobic radicals that may be in accessory chains of protein amino acids. Therefore, the new technique is more reliable and of greater accuracy.

## One-dimensional gel electrophoresis: SDS-PAGE

**Figure 4.**
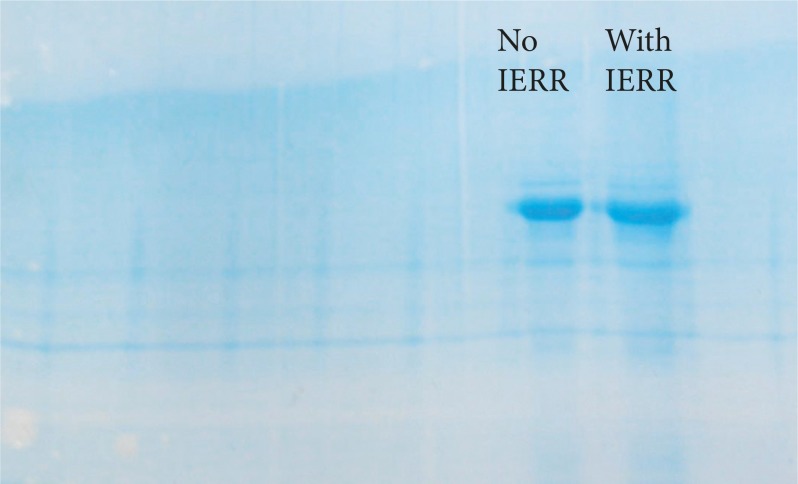
12% SDS-PAGE gel for the GCF of patients with and without IERR obtained by means of the new extraction method.

Analysis of the composition of gingival crevicular fluid proteins in patients with IERR was performed by denaturant electrophoresis in 12% polyacrylamide SDS-PAGE gel at room temperature (Fig 4) and as described by Kojima *et al*.^10^ Invitrogen (Bench Marcker, Protein Ladder) protein markers were used. Subsequently, the gel was stained with Coomassie G-250 brilliant blue.

## Isoelectric focusing

The first dimension was performed at 15^o^C, in Ettan IPGphor 3 (GE Healthcare) appliance, following the manufacturer's instructions (GE Healthcare) and under the following conditions: 500 V for one hour, 1000 V for two hours, 1000 V gradient at 8000 V up to one hour and forty minutes, 8000 V for five hours, totaling 50,000 V/H with upper limit of electric current of 50 mA and potential of 5 W totaling nine hours and 40 minutes. After isoelectric focusing, the strips were stored at -80 °C until the second dimension was carried out.

## RESULTS

**Figure 5.**
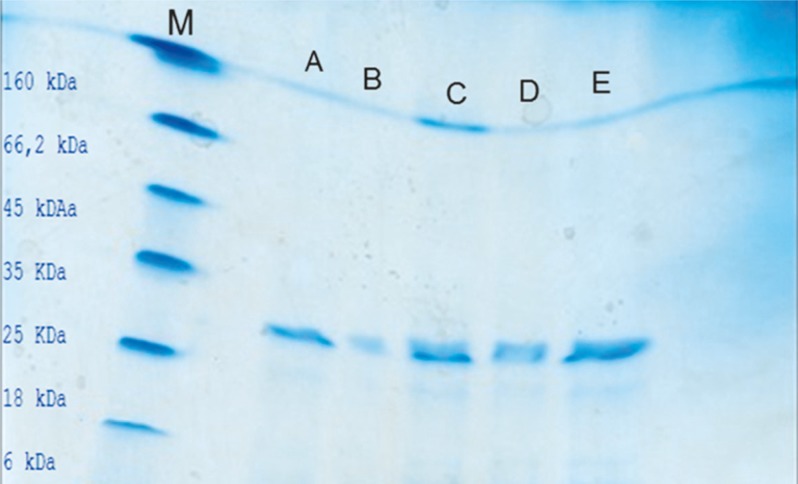
One-dimensional 12% SDS-PAGE gel. Protein extraction by means of sodium phosphate buffer solution stained with Coomassie G-250 blue. M: Molecular marker; A,C,E: mild to moderate external root resorption groups; B,D: groups without external root resorption. Note the traces of salt. 16 µg of samples were applied to each pool of gel.

**Figure 6.**
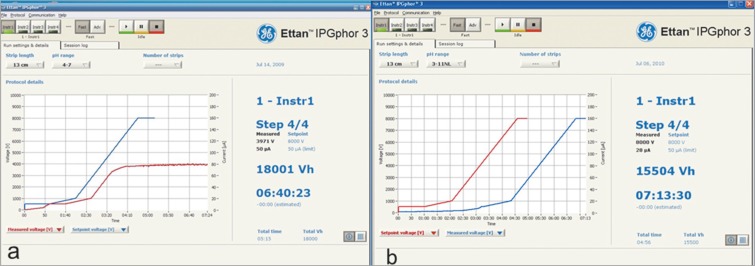
A) Electrophoretic patterns of gel with phosphate buffer; difficulty in carrying out the first dimension due to traces of salt which affected isoelectric focusing. B) Electrophoretic profile with three strips: one control and two strips embedded into two different samples wherein isoelectric focusing carried out by means of the new protein extraction method proved successful.

The gel performing protein extraction by means of sodium phosphate buffer solution (Fig 5) showed traces of salt and did not achieve the first dimension of isoelectric focusing (Fig 6) in the two-dimensional electrophoresis.

**Figure 7.**
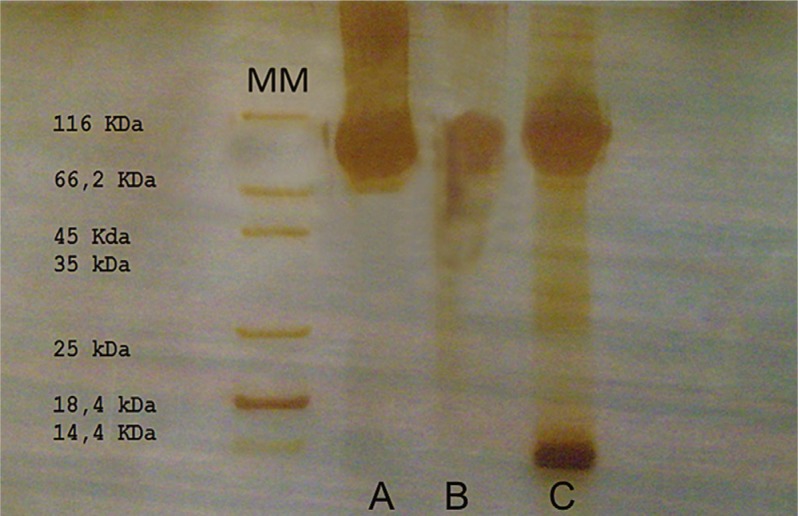
12% SDS-PAGE one-dimensional gel stained with silver nitrate. GCF proteins of different protein precipitation methods. MM, molecular marker; A, IERR sample precipitated with ammonium acetate/dialysis/lyophilization; B, sample without IERR precipitated with ammonium acetate/ dialysis/lyophilization; C, sample without IERR precipitated with TCA/acetone, 50 µl of the lyophilized sample was applied to each pool.

Several classic methods of protein extraction were used, namely: ammonium acetate precipitation with and without dialysis, as well as precipitation by trichloroacetic acid and acetone (TCA acetone). However, in either one of the methods (Fig 7), the resolution of protein bands was satisfactory in terms of sharpness and amount of bands. Additionally, they interfered in isoelectric focusing (during the first dimension) (Fig 6).

Protein extraction without precipitation, but by means of Milli-Q ultrapure ice cold water, was the only protein extraction method that did not interfere in isoelectric focusing during the first dimension of 2-DE gels (Fig 6). Moreover, this method presented better resolution in terms of size and quantity of protein bands, which differs from other techniques.

## DISCUSSION

According to Wellington *Rody,* 2 the dental procedure used to collect samples of GCF consists in removing subgingival plaque (prophylaxis) with a plastic scraper without touching the gingiva, followed by careful drying of the gingiva for 10 seconds with compressed air (syringe) and relative isolation of saliva with cotton rolls. Subsequently, the strips are inserted into the gingival sulcus of teeth for 30 to 60 seconds. The strips are labelled and taken to Eppendorf tubes with phosphate saline buffer, and immediately sent to a laboratory where they are agitated for 15 seconds at room temperature, and centrifuged for 5 minutes at 3.000 g so as to eliminate bacterial biofilm and cellular elements. The supernatant is then stored at -80 °C for further analysis as a biomarker.^11^

This protein extraction method was used by all authors who have studied external root resorption through SDS-PAGE gel; however, the presence of salt in the sample solution (even if dialyzed) hinders isoelectric focusing in the first dimension, when two-dimensional electrophoresis is used. This hampers analysis of electrophoretic and protein profile of the sample (Fig 6).

The presence of contaminants, even after constant washing and dialysis of several methods of protein extraction, probably prevented proper focusing of samples during the first dimension phase. Even with the use of the new method for protein extraction (Milli-Q ultrapure ice cold water), focusing was only possible with increased IPG-Buffer (ampholyte) which is usually used at 0.5%. The use of 2% IPG-Buffer is required to increase conductivity of electrical current during the focusing process, thereby increasing voltage and allowing it to take place normally (Fig 6).

During orthodontic movement, the cementum is reabsorbed and subsequently repaired. Moreover, the products of cementum degradation in the GCF were detected by most researches in both control and treatment groups. Thus, cementum proteins may not be indicative of permanent loss of root structure, which somehow disqualifies them as IERR markers.^5^^,^ 12 ^,^ 13

Although small areas of dentin resorption have been proved to undergo repair, larger apical areas do not undergo repair, thereby rendering dentin loss significant in root structure loss.^5^

Laura Balducci *et al* 4 identified and quantified dentin extracellular matrix proteins: dentin matrix protein 1 (DMP1), dentin phosphoprotein (DPP) and dentin sialoprotein (DSP) in gingival crevicular fluid of individuals undergoing orthodontic treatment.

DMP-1, a non-collagen protein found in dentin and bone mineral matrix, was found in large amounts in the gingival crevicular fluid during the process of resorption. This can be attributed to the presence of this protein which is removed from bone and dentin during IERR. By means of SDS-PAGE gel, Balducci et al^4^ proved that the total concentration of protein in the IERR group was greater than that of the control group, particularly due to degradation of protein matrix during IERR.

In Western Blot analysis, the size of bands was equal in both groups, but more intense for the IERR group.^4^ In the enzyme-linked immunosorbent assay (ELISA), the distribution of values occurred with normal concentrations (DMP-1, DPP, DSP) within all groups; however, the DMP-1 antibody showed high concentrations in the IERR group in comparison to the control group, which did not occur among groups with mild to moderate and severe IERR.^4^

Although there is statistically significant difference between the control and the study group (IERR), DMP-1 is not specific to the dentin and its presence is not only due to the IERR process, but also due to increased bone remodeling during orthodontic movement.^12^ DMP-1 is not a good root resorption marker, since it does not allow us to distinguish between normal and pathological activity.^4^

Balducci *et al* 4 also studied DPP and DSP proteins, and found larger concentrations of DPP and DSP in the severe IERR group, followed by the mild IERR group and the control group. However, the authors used polyclonal antibodies that react to proteins with similar epitopes (antigenic recognition sites), and may indicate the presence of small amounts of DPP and DSP in the control group.^14^ Even so, the authors suggest that since concentrations found in severe and mild to moderate IERR were statistically different, including in comparison with the control group, DPP and DSP might be considered as molecular markers for early detection and dynamic monitoring of IERR. 4

Mah and Neelanjani^5^ related IERR-treated groups and control groups by means of enzyme-linked immunosorbent assay (ELISA), and also found small amounts of DPP in the control group. The authors suggested that this may be due to the high sensitivity of the ELISA method, even if the antibody used for this purpose was developed with DPP of rats - counterparts of human beings. Finding human antibodies for DPP is a challenge due to protein folding and extensive post-translational changes that affect the molecule which, in turn, is shielded by many phosphate and carbohydrate groups.^5^ These phosphate groups are commonly found in other proteins and are not particularly antigenic, thereby hindering the production of human antibodies against DPP.

In addition, the experimental group used by the authors comprised individuals aged between 12 and 16 years old. That is the period when the apex of maxillary incisors is formed (rizogenesis), with odontoblasts and odontoclasts working similarly to osteoblasts and osteoclasts, thereby forming, reabsorbing, remodeling and maintaining dentin.^5^ Dentin remodeling has not yet been proved; however, some researches have demonstrated that dentin tissue is not homogeneous and protein components change with age and root maturation.^15^

Shalene *et al* 6 also found DSP in the control group, but by means of Western Blot. They believed DSP was related to complex structural and cell changes happening within the periodontium, which involved the front of mineralization when root maturation takes shape. They also cited that the basal turnover of dentin matrix proteins occurs during the process of root structuring from deciduous to adult dentition, and that DSP may have been released from the pulp cells when the apex of teeth were still open.^6^^,^ 16

Nevertheless, a research conducted by Quin *et al* 17 may explain the presence of these proteins (DSP, DPP) in the control group of all aforementioned researches. DSP and DPP were transcribed in a single RNA messenger, a transcript derived from a large precursor protein known as DSPP, traditionally considered to be specific to the dentin. The authors found that the DSPP gene was also expressed in osteoblastic cells. DSP was detected in the extracts of long bones of rats in the ratio of 1: 400 in relation to the dentin.^17^ By means of polymerase chain reaction of reverse transcriptase and primers specific to the 5' DSP portion and the 3' DPP sequence, DSPP mRNA was detected in osteoblastic-like cells and osteoblasts of rat calvarium, even if this gene was expressed in a much lower level in dentin osteoblasts than odontoblasts. 6 ^,^ 16

This may indicate that different regulatory mechanisms control the expression of DSPP and are involved in bone tissue and dentin.^6^

The literature does not reach a consensus regarding DPP and DSP proteins as molecular markers due to the presence of these proteins in control groups (even if in small quantities). Evidence shows that these proteins are not unique to the dentin, but are also expressed in bone tissue. Moreover, they might be present in gingival crevicular fluid due to the physiological process of bone remodeling, which is typically increased in patients undergoing orthodontic treatment, and not due to root resorption.^4^^,^ 5 ^,^ 6 ^,^ 14 ^,^ 17

In addition, some studies suggest that dentin remodeling does occur. Furthermore, they also suggest that the dentin is not a homogeneous tissue and its protein components change with age and root maturation even if significant dentin repair does not occur, thereby leading to significant dentin loss.^5^^,^ 15 Thus, it is possible that these alleged proteins be present in gingival crevicular fluid in the absence of disease or not be present in GCF due to tissue aging process.

Some authors believe in the use of these proteins as molecular markers. According to them, logical argumentation is based on the characteristics of the immune system which does not recognize the global structure of proteins, but discrete sites known as epitopes. Large molecules with more than 10 kDa feature a greater number of epitopes capable of potentially increasing the existence of receptors for some of these determinants in lymphocyte cells.^18^ Antigen molecular complexity also increases antigenicity (for instance, with an aromatic ring possessing above three amino acids) which enables DSP (55 kDa) and DPP (140 kDa) dentin proteins of high mass and molecular complexity to be considered as good candidates for antigenic determinants.^18^

The higher the phylogenetic distance between the receiver and the antigen, the greater the antigenicity. Although it does not occur in recognition of dentin (DSP and DPP) by lymphocytes, because both are human, they have sizes and molecular complexity conducive to good immune recognition, including DPP with its extensive post-translational changes that interfere in antigenicity.^19^ The largest post-translational change in DPP is in the phosphate groups which are essential for dental biomineralization.^20^^,^ 21

It is known that inorganic substances never activate lymphocytes, and dentin is covered with hydroxyapatite, totaling 50% of its total weight. Suppa et al^22^ compared the antigenicity of secondary dentin (affected by caries) and normal dentin by means of highly specific monoclonal antibodies. They floated the possibility of protein epitopes being masked by mineral apatite in the region of hyper-mineralized peritubular secondary dentin.^22^ In fact, they found decreased antigenicity, and later found this to be due to denaturation of protein components, thereby disabling identification by the antibody used in the research, with high specificity to intact molecule.^22^

Perhaps, differential mineralization among different individuals and in certain dentin areas is responsible for the extensive variations in IERR presented in the literature, which hinders the presentation of these antigens to the immune system, as it does not recognize the global structure of proteins, but discrete sites known as epitopes.^18^ In this case, the spatial conformation of DPP protein, a post-translational feature, could not only manifest as a sub-clinical deficiency, since differences in detectable situations occur at clinical level, but also cause antigenicity to vary among individuals.

Thus, we could establish risk groups for IERR based on post-translational variations of DPP if the latter was correlated with haplotypes for DPP, since these haplotypes exist in large quantities for this protein^23^ and are seen in the normal population as single silent nucleotide polymorphisms (SNPs). In other words, extensive variations in SNPs for DDP and its alleged post-translational modifications could be correlated. There could also exist some correlation with the degree of IERR, whether affecting or not dentin biomineralization at the molecular level, but not to the clinical one.

In 2007, Kimchi-Sarfaty et al^24^ found SNPs which do not alter the genetic code, but change the function of the protein in which they occur This was reported in the gene of multidrug resistance - and the change of its product: P-glycoprotein (P-gp), which results in changes in the inhibition to drugs. This was explained by conformational changes, with the hypothesis that SNPs alter the time of cotranslational protein folding and P-gp insertion within the membrane, thereby altering the structure of the substrate and the sites of inhibition.

In an insight about the subject, Komar^25^ reported that despite the fact that the codon was degenerated, meaning that many amino acids are represented by more than a triple nucleotide (and these codons are synonymous with respect to translational process), these SNPs are considered silent. This changes the composition of the constituent amino acids of the protein they refer to, and there is no discernible effect on the function of the gene or on the phenotype, there is a change in mRNA translation kinetics in ribosomes, which leads to changes in the final protein structure ("folding") and, therefore, in its function.^25^ The author concludes that SNPs mufflers can contribute to the development and progress of certain diseases.^25^

## CONCLUSION

To date, molecular diagnostic kits for detection of IERR at the clinical level have not yet been developed. No consensus has been reached on the use of these dentin proteins as IERR markers. Further high-resolution protein research methods searching for new molecular markers are still necessary. Two-dimensional electrophoresis followed by mass spectrometry (MALDI-TOF) is the technique of choice for this task.

This new protein extraction technique opens up the possibility to use two-dimensional electrophoresis, since the traditional extraction method used by several authors does not allow isoelectric focusing, necessary for 2-DE gels, to be carried out.
